# Maize response to endophytic *Metarhizium robertsii* is altered by water stress

**DOI:** 10.1371/journal.pone.0289143

**Published:** 2023-11-27

**Authors:** Hannah Peterson, Imtiaz Ahmad, Mary E. Barbercheck

**Affiliations:** Department of Entomology, The Pennsylvania State University, University Park, PA, United States of America; Universitat Jaume 1, SPAIN

## Abstract

To defend against damage from environmental stress, plants have evolved strategies to respond to stress efficiently. One such strategy includes forming mutualist relationships with endophytes which confer stress-alleviating plant defensive and growth promoting effects. *Metarhizium robertsii* is an entomopathogen and plant-protective and growth-promoting endophyte. To determine the context dependency of the relationship between *M*. *robertsii* and maize, we conducted a greenhouse experiment that imposed stress as deficit and excess soil moisture on maize plants which were inoculated or not inoculated with *M*. *robertsii* and measured plant growth and defense indicators. Maize height and endophytic root colonization by *M*. *robertsii* were positively correlated in the deficit water treatment, but not in the adequate or excess water treatments. The relative expression of *ZmLOX1 i*n the jasmonic acid (JA) biosynthesis pathway was significantly greater in *M*. *robertsii*-inoculated than in non-inoculated plants, but water treatment had no effect. There was significant interaction between *M*. *robertsii* and water treatments on foliar concentrations of JA and jasmonoyl isoleucine (JA-ILE), suggesting that water stress impacts *M*. *robertsii* as a modulator of plant defense. Water stress, but not inoculation with *M*. *robertsii*, had a significant effect on the expression of *MYB* (p = 0.021) and foliar concentrations of abscisic acid (p<0.001), two signaling molecules associated with abiotic stress response. This study contributes toward understanding the highly sophisticated stress response signaling network and context dependency of endophytic mutualisms in crops.

## Introduction

Abiotic stressors, such as excess or deficit water, excess salinity, and nutrient deficiency are major limiting factors of plant growth in agricultural systems [1, 2]. To defend against damage from these stressors, a number of beneficial relationships involving plants and soil microorganisms have evolved. Through these relationships, plant hosts generally benefit through plant growth promotion [3], nutrient transfer [4], modulation of plant defenses [5], and suppression of plant disease [6] and herbivorous insects [7]. Among these microorganisms, entomopathogenic fungi (EPF) in the genus *Metarhizium* Sorokin (Hypocreales: Clavicipitaceae), when functioning as an endophyte, are known to confer these benefits to a wide range of plant species. *Metarhizium* spp. have been reported to colonize agriculturally and economically important species including maize [3], tomato [8], haricot bean [9], soybean [10], wheat [4], potato [11], cassava [12], cereal rye [5], Austrian winter pea [5], and sweet pepper [13]. *Metarhizium* spp. have demonstrated a preference for plant roots over above-ground plant tissues [14].

Endophytic colonization by *M*. *robertsii* J.F. Bisch., Rehner & Humber [15] can benefit plant hosts in a variety of ways. Elena et al. [8] showed that tomato plants colonized by *M*. *anisopliae* exhibited significantly greater height and root and shoot length compared with uncolonized plants [8]. Since then, many studies have observed similar results with several different host plants [3, 5, 9, 10, 11]. These benefits have been attributed to several different growth-promoting mechanisms, including the modulation of host plant molecular defenses [5, 16, 17]. The jasmonic acid (JA) pathway is directly related to biotic stress in plants and is mainly associated with biotrophic phytopathogens and phloem-feeding insects [18–22]. Studies investigating the effect of colonization by *M*. *robertsii* on plant defense gene expression found that colonized plants were primed to respond to environmental stressors epigenetically in the JA pathway [5]. This aligns with other studies which reported greater stress tolerance in colonized plants, including salt stress and disease, compared to those that were not colonized [10, 23].

*ZmLox1* is a maize gene in the lipoxygenase (*LOX)* gene family [24, 25]. Oxylipins produced by lipoxygenases are one of many products of the JA pathway and play a key role in defending maize from biotic stressors, including disease and herbivory. Oxylipins can function in many ways, including acting as a signaling molecule, conferring antibacterial and wound protecting properties, and producing cutin, the protective outer layer of the plant [25–27]. Through these physiological responses, a plant can better defend itself from future biotic stressors [19, 26].

*MYB* genes are a well-studied gene family which primarily code for transcription factors which ameliorate various abiotic stresses, including water stress [28]. Previous studies have shown that manipulating the transcription levels of *MYB* genes can increase abiotic stress tolerance by activating stress defense signal transduction pathways, primarily the ABA pathway [29–32]. The JA and ABA pathways have been shown to function synergistically. ABA production is known to stimulate JA production, and vice versa [33, 34].

There are still many gaps in knowledge regarding the multifunctional lifestyle of *M*. *robertsii* and how abiotic factors affect the interaction between endophytes and their host plants. In agricultural production, adverse weather conditions are common and a major obstacle to attaining potential maximum yields. Furthermore, stress from prolonged adverse weather, such as drought and flooding, will be exacerbated due to climate change in many regions [35–37]. Accurate assessment of the mutually beneficial relationship between *M*. *robertsii* and plant hosts will be improved by consideration of how the relationship functions under conditions of abiotic stress [38].

Here we report the results of a greenhouse experiment to compare the effects of the interaction of endophytic colonization and deficit, adequate, and excess soil moisture on the response of maize, *Zea mays* L. We hypothesized that plants subjected to water deficit or excess and colonized by *M*. *robertsii* will have greater height, biomass, and chlorophyll content than non-colonized plants subjected to water stress. To explore the interacting effects of endophytic colonization and water stress on plant growth promoting effects on the molecular level, we analyzed samples from control and test plants for gene expression in the JA, SA, and ABA pathways and content of stress-related phytohormones. We hypothesized that endophytic colonization by *M*. *robertsii* and water stress will alter expression of plant defense genes for pathways associated with defense against biotic and abiotic stress, and subsequently, there will be differential phytohormone content in colonized and uncolonized plants.

## Materials and methods

### Production of *M*. *robertsii* inoculum

To produce inoculum for the experiment, we used an isolate of *M*. *robertsii* from a field experiment to determine the effects of crop species on the prevalence of *Metarhizium* spp. [5, 39] and stored on beads in cryovials at –80°C (Pro-Lab Diagnostics Microbank™ Bacterial and Fungal Preservation System, Toronto). We transferred beads containing spores of *M*. *robertsii* from cryovials to plates containing CTC medium [40] and incubated plates at 25 ± 2°C in the dark for 14 days. We harvested the conidia under aseptic conditions and suspended them in a sterile 0.05% aqueous solution (v/v) of Triton™ X-100 (Dow Chemical Co., Midland, MI). We homogenized the conidial suspension by shaking for one minute, then filtered the homogenized conidial suspension through four layers of sterile cheese cloth to separate the fungal mycelium fragments from conidia. We determined the concentration of the stock conidial suspension under a compound microscope at 400X magnification with a Neubauer hemocytometer and adjusted the concentration to 1×10^8^ conidia ml^-1^ for use in experiments. To confirm the viability of the conidia applied to experimental plants, we plated 80 μl of the conidial suspension onto CTC medium and stored it in dark at 25 ± 2°C for 24 h. We assessed the percent viability by randomly counting 200 conidia at 400X magnification and considered conidia viable if hyphae were visible or the germ tube was at least twice the length of the conidium and only proceeded with the experiment when conidial suspensions had a germination rate of greater than 90%. Spore viability was adequate in all replicates of the experiment.

### Plant growth medium and inoculation

Plant growth medium comprised of field soil and potting mix (Vigoro Industries, Inc., Northbrook, IL) in a 1:1 ratio (v/v) was prepared using the methods described in Ahmad et al. [5]. Plant growth medium was steamed twice for two hours at 121°C in a steam sterilizer to reduce the prevalence of other microbes [5]. Plastic 22.8 cm pots were filled with 5.5L of steamed growth medium and placed randomly on a greenhouse floor with 16L:8D photoperiod at 25 ± 3°C.

To prepare seeds for the experiment, we surface sterilized seeds (*Zea mays* var. 4050, Master’s Choice, organic) in a sterile laminar flow hood by immersion in 0.5% sodium hypochlorite for two minutes followed by soaking in 70% ethanol for two minutes and rinsing three times in sterile distilled water [41]. Once the seeds were dry, we planted one seed directly into the prepared pots by using sterile spatulas to a depth of ~2.5 cm in each pot. Plants were then grown with adequate water for 14 days.

### Treatment groups

Following the initial 14-day period to allow for seed germination, plants were divided evenly into the following treatment groups: 1) Deficit moisture + *M*. *robertsii*, 2) deficit moisture, no *M*. *robertsii*, 3) adequate moisture + *M*. *robertsii*, 4) adequate moisture, no *M*. *robertsii*, 5) excess moisture + *M*. *robertsii*, 6) excess moisture, no *M*. *robertsii*. To inoculate pots in treatments with *M*. *robertsii* we applied 12 ml of fresh conidial suspension directly to the soil at the base of each test plant. Pots in treatments without inoculation of *M*. *robertsii* were inoculated with 12 ml of 0.05% aqueous solution (v/v) of Triton™ X-100.

Soil in deficit moisture treatments was maintained below 10% soil moisture content, which was measured daily using an ML3 Thetaprobe (Dynamax Inc., Houston, Texas) soil moisture meter. Soil in the excess moisture treatments was maintained between 30–40% soil moisture content. Excess moisture treatments were maintained at high soil moisture content by placing plastic pans placed under the pot to prevent water drainage and create waterlogging conditions. Soil in the adequate moisture treatments was maintained between 15–25% soil moisture. Each experiment ran for a total of 5 weeks: two weeks of initial maize growth with adequate soil moisture followed by three weeks in which water and *M*. *robertsii* treatments were imposed. Three trials were conducted, and there were 9, 8, and 10 plants in each treatment group, respectively, for a total of 486 plants.

### Plant response

After applying water stress to plants for 21 days, maize plants were nondestructively sampled for plant height, chlorophyll content, and infrared temperature one day before destructive harvest. We measured plant height (cm) from the base of the plant to the tip of the longest fully emerged true leaf at V4 stage. We measured total chlorophyll content of the fourth true leaf (SPAD 502 Plus Chlorophyll Meter, Spectrum Technologies, Inc., Aurora, IL) from three sections of the same fourth true leaf: the portion closest to the leaf node, the center of the leaf, and the portion near the tip of the leaf, and calculated the mean of the readings. We measured temperature from the same leaf and in the same location as chlorophyll content (ISO 17025 Calibrated Infrared Thermometer, Traceable Products, Webster, Texas). Between each plant, we wiped the ruler, thermometer, and chlorophyll meter with 70% ethanol using Kimwipes™ to avoid contamination. At the end of the experiment, we measured above-ground biomass by cutting the plant at the soil–plant interface with clean scissors, excluding the fourth true leaf. We placed the maize biomass in dried, pre-weighed brown paper bags, and oven-dried them at 60°C for 10–14 days, when we weighed the biomass using a digital balance.

### Detection of endophytic colonization

At the end of the experiment, we evaluated the endophytic colonization of maize by *M*. *robertsii*, when the plants were at approximately V7 [42]. From each plant, we removed the fourth true leaf and one 10-cm long primary root sections and rinsed excised roots with tap water to remove soil. We surface sterilized the excised leaf and root sections by submerging in 0.5% sodium hypochlorite for three minutes followed by 70% ethanol for two minutes, followed by serially rinsing three times in sterile deionized water. These solutions were changed between treatments to prevent contamination. To confirm tissue sterilization, we plated 80 μl of the final rinse water onto Sabaroud Dextrose Agar (SDA) medium and kept the dishes at 25 ± 2°C for 10 days in darkness. We cut off ∼1 mm of outer edges of the surface sterilized leaf and ends of the root tissues using sterile dissecting scissors to remove dead cells. We cut each leaf into six, 6 × 6 mm sections and each root section into six, 6 mm long sections so that each plant generated six leaf and six root sections. We plated each tissue type from each plant in a labeled petri dish prepared with CTC medium by pressing the tissue flat against the surface of the medium. The plates were sealed with parafilm and incubated in the dark at 25 ± 2°C for 14 days. We identified *M*. *robertsii* by its characteristic white hyphal growth and dark green conidia and confirmed identity as *M*. *robertsii* by the methods of Kepler et al. [43].

In total, we plated 486 root and 486 leaf sections from 81 *M*. *robertsii*-inoculated plants and 486 root and 486 leaf sections from 81 control plants. 27 plants each were from *M*. *robertsii*-inoculated treatments from the deficit, adequate, and excess moisture treatments, respectively. The same number was obtained from non-inoculated treatments. We considered a plant to be endophytically colonized when we observed growth of *M*. *robertsii* from one or more root or leaf sections. We calculated proportion endophytic colonization of plants among water treatments by dividing total number of plants with root, leaf or both root and leaf colonization within each water treatment group by total number of *M*. *robertsii*-inoculated plants. Intensity of colonization was calculated from the mean proportion of colonized tissue sections collected from *M*. *robertsii*-treated plants.

### Gene expression analysis and phytohormone profiles

We removed a 15 mm section of the fourth true leaf from each maize plant for analysis of defense gene expression and phytohormone content. We then removed approximately ~100–150 mg of the fourth true leaf and placed it into pre-labeled 2 ml Eppendorf tube, flash froze the tube in liquid nitrogen, and then stored at -80°C until processing for gene expression and phytohormone content. To extract RNA from the frozen tissue from the 4th true leaf of control (non-inoculated) and *M*. *robertsii*-inoculated V7 maize plants, we homogenized 0.1 g of the leaf tissue in liquid nitrogen (GenoGrinder 2000, OPS Diagnostics) followed by extraction with 1 ml of TRIzol (Life Technologies, USA) per ∼ 0.1 g of tissue. We quantified the genomic DNA-free RNA (Nanodrop, Thermo-Fisher Scientific), and used 1 μg of total RNA to make complementary DNA (cDNA) (High Capacity cDNA Reverse Transcription kit, Applied Biosystems) and oligo(dT). Then, we performed qRT-PCR (7500 Fast Real-Time qPCR, Applied Biosystems, ThermoFisher Scientific, Inc.) with Fast Start Universal SYBR Green Master Mix (Roche Molecular Systems, Inc.) with actin as a reference gene and gene-specific primers ([24]; Table 1). The target genes we used in this study were *lipoxygenase 1* (*ZmLOX1*) and *Myeloblastosis 3R (ZmMYB3R*, Table 1)

**Table 1:** Forward and reverse primer sequences for the genes tested in this study and actin (endogenous control).

Gene	Forward Primer	Reverse Primer	Number	Reference
**Actin**	GGAGCTCGAGAATGCCAAGAGCAG	GGAGCTCGAGAATGCCAAGAGCAG	U60511.1	Moniz & Drouin, 1996 [24]
**Lipoxygenase 1** ***ZmLOX1***	CGTTCCGTGAAGTGTGGTTCT	CGTTCCGTGAAGTGTGGTTCT	AF271894	Shivaji et al., 2010 [44]
**Myeloblastosis 3R** ***ZmMYB3R***	GTCCGTGACAAGGACCAAGAA	ACCCCAACAGGATCAGGTGTT		Wu et al., 2019 [30]

We conducted the qRT-PCR using the default conditions: 50°C for 2 min and 95°C for 10 min; 95°C for 30 s and 60°C for 1 min repeated for 35 cycles; 72°C for 10 min; and dissociation stage. We performed RNA extraction, cDNA synthesis, and qRT-PCR for each biological replicate separately, and each treatment and block had two biological replicates. In total, there were 36 biological replicates. There were three technical replicates, three endogenous controls, and three negative controls per biological replicate in each round of qRT-PCR.

The phytohormone profiling of pre-weighed maize leaf tissue was performed by the Proteomic and Metabolomic Facility of The Nebraska Center for Biotechnology at The University of Nebraska, Lincoln. The content of the following phytohormones were determined in each sample by HPLC/MS: abscisic acid (ABA), jasmonic acid (JA), jasmonoyl-isoleucine (JA-ILE), 12-oxophytodienoic acid (OPDA), salicylic acid (SA), 2,4-dihydroxy-7-methoxy-1,4-benzoxazin-3-one (DIMBOA), cis-zeatin (cZ), cis-zeatin riboside (cZR), gibberellin 19 (GA19), gibberellin 53 (GA53), indole-3-acetic acid (IAA), methylated indole-3-acetic acid (MethylIAA), and strigol. Here we report results only for phytohormone content that differed significantly among experimental treatments.

### Data analysis

We analyzed all data in R Version 3.6.1 [45] unless otherwise stated. We used multiple linear regression (function lm) in the base R package to determine the effect of water treatment and maize root colonization on maize height, biomass, infrared leaf temperature, chlorophyll content, and relative water content. We designated all treatment variables as fixed factors and block (trial number) as a random factor. We used simple linear regression (function lm) for each water treatment to determine the effect of intensity of maize root colonization on maize height, biomass, infrared leaf temperature, chlorophyll content, and relative water content. We considered the results of analyses significant at p<0.05. For all analyses, we transformed proportions using square root arcsine transformation (ArcSin function) to meet assumptions of normality, equality of variances and to reduce heterogeneity of variances. Data presented in figures are not transformed. Figures were created using the packages patchwork, dplyr, ggplot2 and reshape2 in R [46–48].

We used two-way ANOVA (function aov) in the base R package to determine the effect of water treatment and maize root colonization on leaf phytohormone content. We designated all treatment variables as fixed factors and block (trial number) as a random factor. We followed the same method for determining the effect of water treatment and maize root colonization on relative expression of *ZmLox1* and *MYB* genes.

## Results

### Endophytic colonization rate

Overall rates of root colonization of maize plant treatments in which *M*. *robertsii* was applied was 33.33 (±0.38) %. Mean root colonization in inoculated deficit, adequate, and excess water treatments was 31±1.09%, 44±1.19%, and 25±1.01%, respectively. Mean leaf colonization in uninoculated deficit, adequate, and excess water treatments was 0%, 0%, and 1.2±1.16%, respectively.

### Relationship between water stress, endophytic colonization, and plant growth

There were no significant differences due to water or *Metarhizium* treatment in foliar chlorophyll content, relative water content, or infrared temperature of maize foliage. Data is available at (https://osf.io/u38ds).

### Height

**Fig 1:**
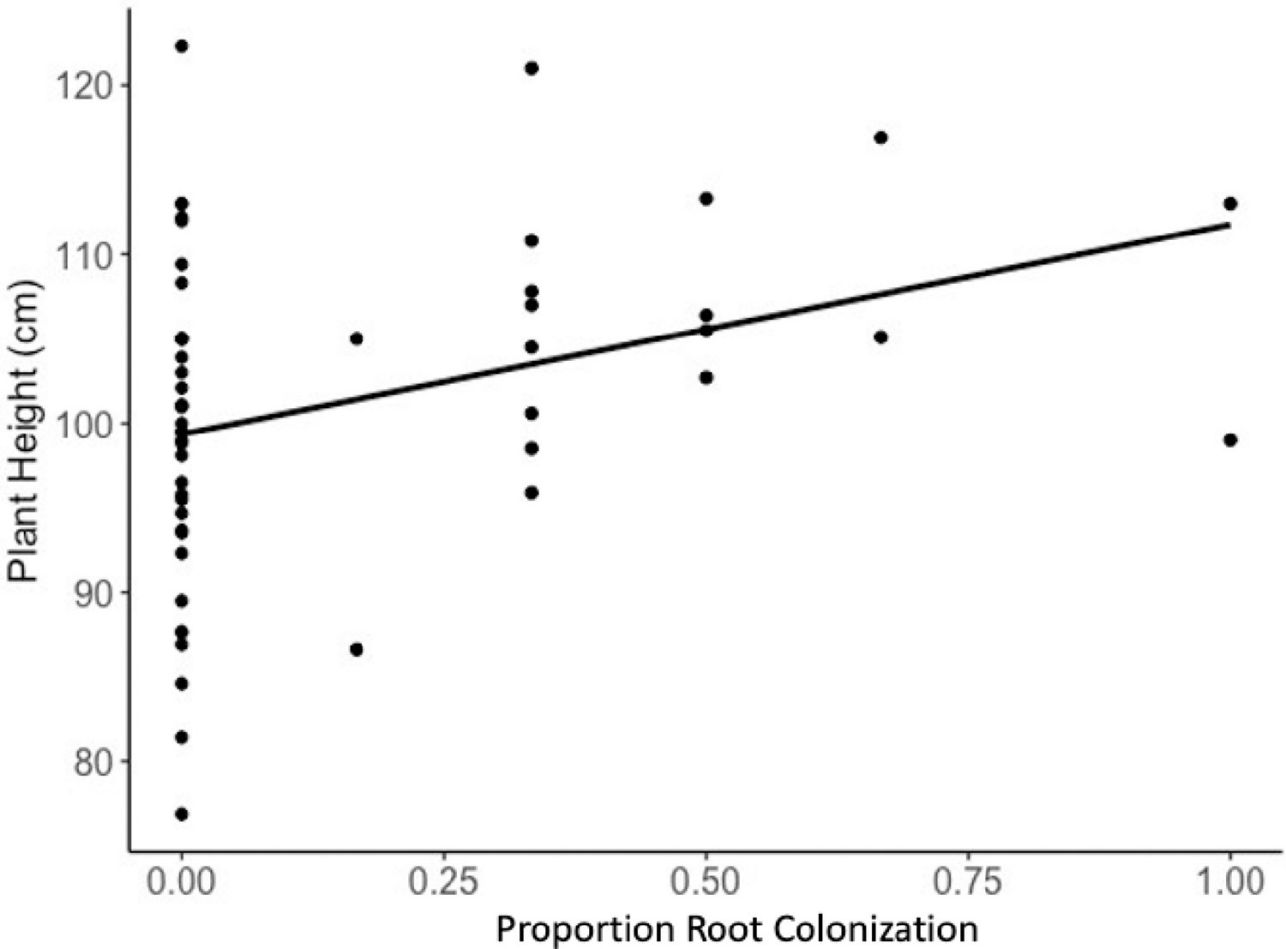
Relationship between intensity of root colonization and plant height among test plants in the deficit water treatment (R2adj = 0.091, *F*_1, 52_ = 6.301, *P* = 0.01).

Among all test plants, water treatment had a significant (*F*_2,2_ = 138.2; *P*<0.0001) effect on maize height. Plants in the excess water treatment (128.1 ± 4.3 cm) were taller than plants in the adequate (123.4 ± 4.3 cm; *P* = 0.0183) and deficit (101.6 ± 4.3 cm, *P*<0.0001) water treatments; and plants in the adequate water treatment were taller (*P*<0.0001) than in the deficit water treatment. Linear regression showed a significant positive relationship between height and intensity of maize root colonization in the deficit water treatment (r^2^_adj_ = 0.091, *F*_1, 52_ = 6.301, *P* = 0.01), but not in the adequate (r^2^_adj_ = -0.008, *F*_1, 51_ = 0.5767, *P* = 0.451, https://osf.io/u38ds) or excess (r^2^_adj_ = 0.019, *F*_1, 51_ = 1.93, *P* = 0.1714, https://osf.io/u38ds) water treatments (Fig 1).

### Biomass

**Fig 2:**
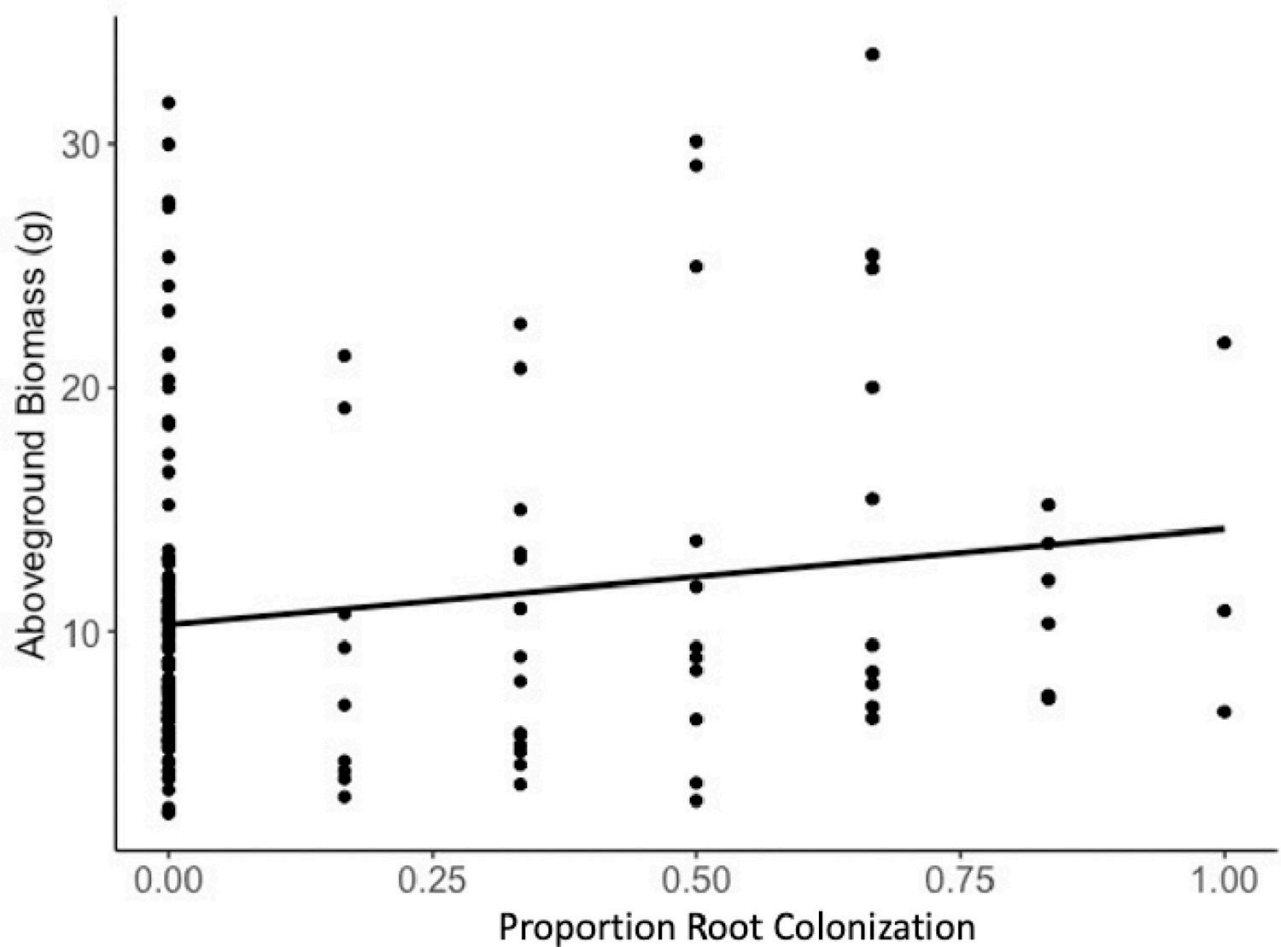
Relationship between intensity of root colonization and plant aboveground biomass across all treatments (r2adj = 0.2008, *F*_3, 152_ = 13.98, *P*<0.001).

Among all test plants, there was a significant positive relationship between maize root colonization intensity and water treatment on plant aboveground biomass (Fig 2; r^2^_adj_ = 0.2008, *F*_3, 152_ = 13.98, *P*<0.001). Biomass was greater *(P =* 0.018) in colonized plants than non-colonized plants and positively related to intensity of maize root colonization *(P =* 0.034, Fig 2) among all test plants. Biomass was also significantly different by water treatment. Average biomass was lower in the deficit water treatment (7.18±0.47 gm, *P*<0.001) than in the adequate (11.74±1.11 gm) and excess (14.43±1.6 gm) water treatments. Simple linear regression of intensity of root colonization within water treatment showed that there was no significant relationship between biomass and maize root colonization intensity in the deficit (r^2^_adj_ = -0.017, *F*_1, 52_ = 0.0697, *P* = 0.7900) or adequate water treatments (r^2^_adj_ = 0.006, *F*_1, 51_ = 1.341, *P* = 0.252). The relationship between biomass and maize root colonization intensity in excess water treatment was marginally significant (r^2^_adj_ = 0.0567, *F*_1, 47_ = 3.885, *P* = 0.054).

### Maize defense gene expression

Relative expression of *ZmLox1* was significantly greater in maize inoculated (12.8 ±2.35) with *M*. *robertsii* compared to maize that was not inoculated (3.95 ±0.94; *F*_2,1_ = 5.849, *P* = 0.017). Water treatment did not have a significant effect on expression of *ZmLox1* (*F*_1,2_ = 1.31, *P* = 0.274), and there was no significant interaction (*F*_2,1_ = 7.93, *P* = 0.4500) between water treatment and *M*. *robertsii* inoculation on expression of *ZmLox1*.

The relative expression of *MYB* was significantly greater in the adequate (1.04 ± 0.10) and deficit (1.05 ± 0.070) water treatments than in the excess (0.74 ±0.080) water treatment (*F*_2,1_ = 4.014, *P* = 0.0210). Inoculation with *M*. *robertsii* did not have a significant effect on relative expression of *MYB* (0.95 ± 0.05; *F*_1,2_ = 0.143, *P* = 0.706), and there was no significant interaction between water treatment and *M*. *robertsii* inoculation on *MYB* expression (*F*_2,1_ = 2.07, *P* = 0.1310).

### Phytohormone profile of maize leaf tissue

There was a significant difference between water treatments in ABA (F_2,1_ = 60.516, *P* <0.0001), cZR (F_2,1_ = 7.645, *P* = 0.0020), DIMBOA (F_2,1_ = 4.782, *P* = 0.0160), and OPDA (F_2,1_ = 3.786, *P* = 0.0340). Tukey HSD post-hoc analysis showed that ABA content was significantly higher in the deficit (380.5758 ± 13.7508) water treatment than in adequate (9.3608 ± 0.6161) and excess (11.195 ± 0.6737) water treatments *(P<*0.0001). Post-hoc analysis revealed that cZR content was also significantly higher in the deficit (4.68 ± 0.3801) water treatment compared to the adequate (1.3825 ± 0.1166, *P* = 0.02) and excess (0.3533 ± 0.0114, *P* = 0.002) water treatments. DIMBOA content was significantly greater in the deficit (1760.73 ± 173.29) water treatment than in adequate (511.22 ± 30.34, *P* = 0.049) and excess (230.47 ± 25.6, *P* = 0.01) water treatments. OPDA content in the adequate (1,054.83 ± 72.62) water treatment was significantly higher than in the excess (454.67 ± 29.91) water treatment *(P =* 0.049) and marginally lower than in the deficit (501.79 ± 31.97) water treatment *(P =* 0.074).

There were significant interaction effects between water and *M*. *robertsii* treatments in JA (*F*_1,2_ = 6.61, *P* = 0.0040) and JA-ILE (*F*_1,2_ = 4.364, *P* = 0.0220) content. A post-hoc Tukey’s HSD test revealed that in the deficit water treatment, JA was significantly lower in plants in treatments inoculated with *M*. *robertsii* (5.84 ± 0.44; *P* = 0.0090) compared to uninoculated (10.63 ± 0.72) controls. Post-hoc analysis revealed no significant interactions between JA-ILE levels among water and *M*. *robertsii* treatments.

## Discussion

### Relationship between water stress, endophytic colonization, and plant growth

Among all test plants, there was a significant positive effect of intensity of root colonization by *M*. *robertsii* on plant height and aboveground biomass, suggesting that *M*. *robertsii* promotes plant growth in maize regardless of water stress level. The results of this study are consistent with other studies which demonstrate a positive effect of *Metarhizium* spp. colonization on plant growth. *Metarhizium* spp. have been shown to increase aboveground biomass and height in maize plants that were not water stressed [5]. Endophytic root colonization by *M*. *robertsii*, *M*. *brunneum*, and *M*. *anisopliae* increased stalk length, leaf collar formation, ear and foliage biomass of maize [3]. In tomato, endophytic root colonization by *M*. *anisopliae* increased plant height, root length, and root and shoot dry weight [8]. The lack of effect of *M*. *robertsii* treatment on foliar chlorophyll content, relative water content, or infrared temperature of maize foliage is consistent with previous results [3, 5, 12].

**Fig 3:**
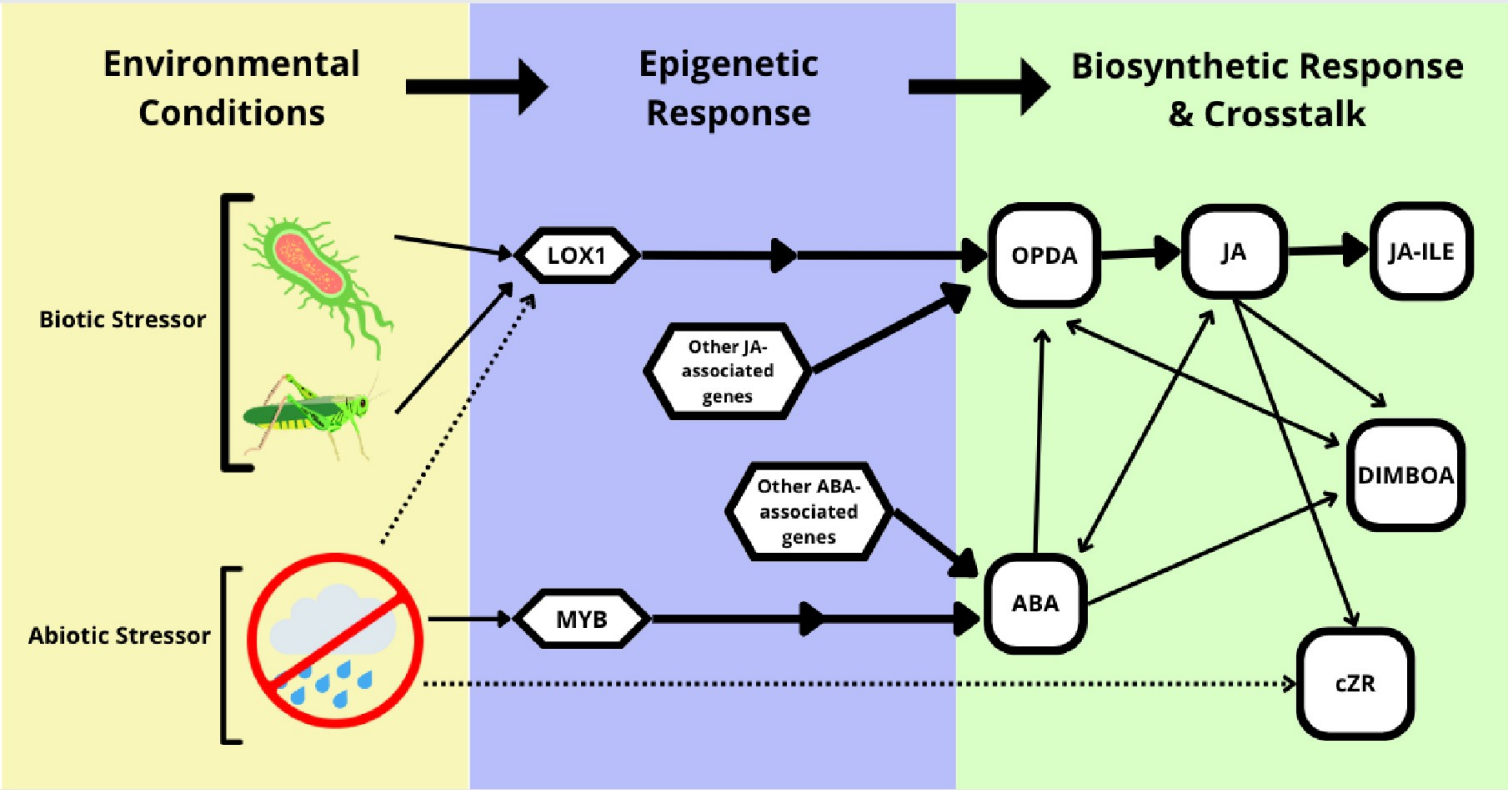
Graphical representation of plant defense pathways discussed in this study. Dotted arrows represent weak or peripheral stimulation in the direction of the arrow, and solid arrows represent strong or central stimulation in the direction of the arrow. Arrows with two points represent synergistic relationships. Multiple successive arrows represent multiple intermediate steps.

Analyzing plant responses within water treatments provided more specific information about the relationship between endophytic colonization, soil moisture, and maize growth. There was a significant and positive relationship between intensity of root colonization and plant height among plants in the deficit water treatment (Fig 1), while no such relationship was significant in the adequate and excess water treatments (Figs 2 and 3, https://osf.io/u38ds). This demonstrates the influence of context-dependent effects on the outcomes of plant-microbe interactions.

Context dependency in a mutualism is defined as the change in interaction net outcome, the magnitude of which can be attributed to any change in the biotic or abiotic environment [49–51]. The change can be positive or negative and is dependent on both the host and endophyte species [10, 52]. There are many examples of context dependent mutualisms involving endophytic fungi and plant hosts. For example, the grass species, *Elymus virginicus* L. had greater aboveground biomass under drought conditions when colonized by the fungal endophyte, *Epichloë elymi* Schardl & Leuchtm., compared to non-colonized hosts [53]. However, the endophyte had a greater positive effect on plant growth when the host plant was not drought-stressed [54]. Other studies have found that the effects of endophytic *Metarhizium* spp. on host plants can be context dependent with changes in environmental conditions. For example, potato plants under nutrient stress were colonized by *M*. *brunneum* at higher rates than non-stressed plants and colonized plants had greater biomass and leaf development than uncolonized plants [11].

### Gene expression

Previous studies have suggested that endophytic entomopathogenic fungi can prime defense gene pathways and increase tolerance to several types of stressors [5, 31]. Some of the most well-known plant defense pathways include the salicylic acid (SA), abscisic acid (ABA), jasmonic acid (JA), and ethylene (ET) pathways. While each pathway is highly associated with certain types of stressors, synergism and antagonism between the pathways can also affect their regulation [54]. In this study, we measured the relative expression of two genes that code for active biomolecules in the ABA and JA pathways [25, 30].

In this study, expression of *ZmLox1*, in the JA biosynthesis pathway, was upregulated in plants that were inoculated with *M*. *robertsii* but were not affected by water stress. Ahmad et al. [5] found similar results where *M*. *robertsii* inoculated plants had greater relative expression of *ZmLox1* compared to non-inoculated plants, and so we expected that inoculation with *M*. *robertsii* would have a significant effect on the expression of this gene. However, this study suggests that water stress conditions do not affect the modulation of *ZmLox1* expression by *M*. *robertsii* in maize.

In our study, only water treatment had a significant effect on relative expression of *MYB*. Relative expression of *MYB* in deficit and adequate treatment was significantly greater than in the excess water treatment. This is consistent with previous reports which have identified the *MYB* gene as a positive transcription factor for drought-related genes [29, 31, 32].

### Phytohormone content

Several phytohormones, such as auxins, gibberellins, and cytokinins can influence water stress tolerance in plants [55, 56]. In this study, there were no significant changes in the pattern of phytohormone content in maize plants based solely on endophytic colonization by *M*. *robertsii* (Table 2). However, there were some significant interactions between water and *M*. *robertsii* treatments. Phytohormones are highly associated with plant growth and defense [57]. The content of four of the phytohormones tested in this study were significantly affected by water treatment: ABA, DIMBOA, OPDA, and cZR (Tables 2 and 3). ABA is associated with the modulation of plant growth and defense against abiotic stress, including water stress [58]. Under water-deficit conditions, ABA is known to initiate water-saving activities such as stomatal closure, increased root growth, and reduced leaf expansion [59]. In this study, the *MYB* gene expression was measured because of its role in the ABA production pathway [28] (Fig 3). *MYB* gene expression in plants in the deficit and adequate water treatment was greater than in the excess water treatment, which supports the current literature that identifies it as a drought responsive gene [32]. Abscisic acid concentration was significantly greater in plants in the deficit water treatment compared to the adequate and excess water treatments *(P <*0.0001). We suggest that cross talk between gene transcription and ABA synthesis intensified the response of plants to drought, resulting in significantly greater ABA concentration in plants in the deficit water treatments (Fig 3). ABA interacts synergistically and antagonistically with other phytohormones such as JA [60, 61]. ABA and JA share multiple signaling genes, such as *MYC2*, which regulates major signaling genes in the JA pathway [33, 34]. We suggest that cross talk in later stages of the ABA pathway resulted in the difference between *MYB* gene expression and ABA production [65].

**Table 2:** Statistical values for phytohormone contents of maize foliage according to water and *Metarhizium* treatment determined by two-way analysis of variance.

Phytohormone	Water Treatment			*Metarhizium* Treatment			Water Treatment*Metarhizium Treatment		
	df	*F*	*P*	df	*F*	*P*	df	*F*	*P*
ABA	2	60.52	<0.001	1	1.18	0.29	2	1.01	0.38
cZ	2	1.71	0.2	1	1.92	0.18	2	0.16	0.86
cZR	2	7.65	0.002	1	0.2	0.66	2	0.53	0.6
DIMBOA	2	4.78	0	1	0.02	0.88	2	0.36	0.7
GA19	2	0.73	0.49	1	0.29	0.6	2	0.35	0.71
GA53	2	0.2	0.82	1	0.17	0.68	2	0.11	0.9
IAA	2	0.68	0.51	1	0.17	0.68	2	0.11	0.9
JA	2	1.39	0.26	1	2.42	0.13	2	6.61	0.004
JA-ILE	2	0.59	0.56	1	0.001	0.98	2	4.36	0.02
OPDA	2	3.79	0.03	1	1.14	0.3	2	0.56	0.58
SA	2	0.58	0.57	1	0.06	0.8	2	0.74	0.48

**Table 3:** Mean ± standard error of the phytohormone contents of maize foliage according to water and *Metarhizium* treatment.

Water Treatment	Adequate		Deficit		Excess	
*Metarhizium* Treatment	Inoculated	Not Inoculated	Inoculated	Not Inoculated	Inoculated	Not Inoculated
ABA	8.91±0.32	9.82±0.25	429.56±5.12	331.587±6.93	14.42±0.36	7.97±0.17
cZ	0.68±0.02	0.81±0.02	0.90±0.03	1.30±0.04	0.42±0.01	0.81±0.02
cZR	1.12±0.05	1.65±0.06	5.56±0.20	3.80±0.14	0.38±0.01	0.33±0.01
DIMBOA	351.03±17.09	671.40±18.19	2036.67±59.97	1484.80±95.36	214.13±7.12	246.82±15.27
GA19	54.73±0.62	49.03±1.12	69.97±1.72	53.83±0.73	44.17±0.87	49.38±1.45
GA53	1.07±0.07	1.75±0.16	0.88±0.08	1.82±0.11	2.24±0.15	1.99±0.13
IAA	12.77±0.05	14.11±0.09	12.24±0.378	9.64±0.34	12.71±0.09	14.22±0.17
JA	2.42±0.03	7.92±0.23	2.38±0.04	20.56±0.68	12.70±0.42	3.42±0.07
JA-ILE	1.16±0.06	1.09±0.04	0.41±0.02	3.88±0.15	3.91±0.19	0.59±0.01
OPDA	900.88±28.54	1208.77±37.4	298.92±4.84	704.67±16.89	494.57±16.4	414.78±10.7
SA	55.90±0.86	89.87±1.79	80.72±2.92	70.60±3.27	124.03±4.5	82.32±2.16

DIMBOA is an anti-insect benzoxazinoid primarily associated with defense against herbivorous insects, but its regulation is not well understood [32]. However, DIMBOA synthesis has been demonstrated to be stimulated by increased concentrations of ABA and JA [62, 63]. In maize in deficit water treatments, DIMBOA concentration was significantly greater than in the adequate or excess water treatments. Belowground increases in ABA, which can be triggered by drought conditions, have been demonstrated to increase aboveground concentrations of DIMBOA [63] (Fig 3). We suggest that the increase in DIMBOA concentration observed in plants in the deficit water treatments in this study was associated with increased levels of ABA.

OPDA is an active biomolecule in the JA biosynthesis pathway and a direct precursor to JA [64]. Accumulation of OPDA and other forms of JA have been associated with higher concentrations of ABA and DIMBOA [54, 62] (Fig 3). However, in this study OPDA was significantly lower in plants in the deficit water treatments compared to the adequate and excess water treatments, while ABA and DIMBOA concentrations were greater. We suggest that this was due to the complexity of plant stress response networks. Whereas phytohormones are known generally to interact synergistically and antagonistically with one another, the context dependency of these interactions makes it difficult to say if two phytohormones will always interact in the same way without exception [6, 65].

cZR (cis-zeatin riboside) is a form of cytokinin (CK), a phytohormone primarily associated with plant growth and development in seedlings and immature plants, particularly in growth limiting conditions [66, 67]. cZR is the riboside of cZ, a signaling molecule in the cytokinin pathway that was also measured in this study [66]. In a previous study, cZR was upregulated in the presence of increased JA concentration, linking it to biotic stressors [67]. Additionally, concentration of cZR has been observed to increase dramatically in plants experiencing drought conditions [66]. This is consistent with the results of this study, where cZR concentration was significantly greater in plants in the deficit water treatments compared to the adequate and excess water treatments. Previous studies have shown that abiotic stressors such as drought, salt stress, and temperature can result in differential production of cZ and cZR, [68–71]. We suggest that concentrations of cZR were significantly different among water treatments because in the deficit water treatment the expression of CK was upregulated, and the effect was intensified due to later crosstalk with the JA pathway (Fig 3). Maize generally has very high levels of cZ and cZR, which suggests that these bioactive molecules are not used only under stress or growth-limiting conditions [72].

Jasmonic acid (JA) and jasmonoyl isoleucine (JA-ILE) were significantly different due to the interacting effects of water and *M*. *robertsii* treatments (Table 2). In the deficit and adequate water treatments, plants inoculated with *M*. *robertsii* had lower concentrations of JA than uninoculated plants. However, plants in the excess water treatment and inoculated with *M*. *robertsii* had higher concentrations of JA than uninoculated plants. Jasmonates are well-known stress hormones that react to several types of stressors [19, 65]. Different forms of JA activate plant defense responses to biotic stressors, such as attack by necrotrophic phytopathogens and chewing insects [5, 19], as well as by abiotic stressors such as salinity [73], drought [74–76], and UV irradiation [77]. Previous studies have found that colonization by *Metarhizium* spp. increases JA concentration in host plants or upregulates defense genes associated with the JA synthesis pathway, which is consistent with the pattern seen in the excess water treatment in this study and gene expression results for *ZmLox1* [5, 10]. However, plants in the deficit water + *M*. *robertsii* treatment had significantly lower concentrations of JA than uninoculated plants. We suggest that the initial molecular response that was observed through upregulation of *ZmLox1* in inoculated plants was triggered by the presence of *M*. *robertsii*, and subsequent hormonal regulation in JA and JA-ILE occurred at the biosynthetic level via crosstalk between phytohormones (Fig 3). While *ZmLox1* is not primarily associated with water stress, we suggest that the central role of JA as a stress regulator in plants and crosstalk with the ABA pathway influenced the effect of water treatment on JA concentration. The difference in phytohormone response to *M*. *robertsii* colonization between water treatments demonstrates how the JA pathway functions differently under different environmental conditions, which may be in part due to the complex negative and positive relationships between JA and other phytohormone pathways [61, 65]. This is ultimately an advantage for plants because complexity among these pathways allows plants to respond to stressors in a targeted manner [78].

## Conclusion

Assessment of the relationship between endophytes and plant hosts will be improved by understanding how the relationship functions under conditions of various types of abiotic stress to determine if crops can be managed to support both agronomic needs and conservation of beneficial organisms. We demonstrated that the plant growth promoting benefits conferred by *M*. *robertsii* to maize hosts are context dependent on soil moisture. The interacting effects of water treatment and maize root colonization intensity on jasmonic acid and jasmonoyl isoleucine demonstrate how water stress can change how *M*. *robertsii* modulates plant defenses. Results such as these will be useful in developing strategies for effective management of *M*. *robertsii* and endophytes in agroecosystems.
